# Weighted Gene Coexpression Network Analysis Identifies TBC1D10C as a New Prognostic Biomarker for Breast Cancer

**DOI:** 10.1155/2022/5259187

**Published:** 2022-04-05

**Authors:** Huiying Qiao, Rong Lv, Yongkui Pang, Zhibing Yao, Xi Zhou, Wei Zhu, Wenqing Zhou

**Affiliations:** ^1^Department of General Practice, Suzhou Ninth People's Hospital, Suzhou, Jiangsu 215200, China; ^2^Department of General Surgery, The Fifth People's Hospital of Wujiang Area, Suzhou, Jiangsu 215211, China; ^3^The Department of General Surgery, Zhongshan Hospital, Fudan University, Shanghai 200032, China

## Abstract

**Background:**

Immune checkpoint inhibitors are a promising therapeutic strategy for breast cancer (BRCA) patients. The tumor microenvironment (TME) can downregulate the immune response to cancer therapy. Our study is aimed at finding a TME-related biomarker to identify patients who might respond to immunotherapy.

**Method:**

We downloaded raw data from several databases including TCGA and MDACC to identify TME hub genes associated with overall survival (OS) and the progression-free interval (PFI) by WGCNA. Correlations between hub genes and either tumor-infiltrating immune cells or immune checkpoints were conducted by ssGSEA.

**Result:**

TME-related green and black modules were selected by WGCNA to further screen hub genes. Random forest and univariate and multivariate Cox regressions were applied to screen hub genes (MYO1G, TBC1D10C, SELPLG, and LRRC15) and construct a nomogram to predict the survival of BRCA patients. The *C*-index for the nomogram was 0.713. A DCA of the predictive model revealed that the net benefit of the nomogram was significantly higher than others and the calibration curve demonstrated a good performance by the nomogram. Only TBC1D10C was correlated with both OS and the PFI (both *p* values < 0.05). TBC1D10C also had a high positive association with tumor-infiltrating immune cells and common immune checkpoints (PD-1, CTLA-4, and TIGIT).

**Conclusion:**

We constructed a TME-related gene signature model to predict the survival probability of BRCA patients. We also identified a hub gene, TBC1D10C, which was correlated with both OS and the PFI and had a high positive association with tumor-infiltrating immune cells and common immune checkpoints. TBC1D10C may be a new biomarker to select patients who may benefit from immunotherapy.

## 1. Introduction

Breast cancer (BRCA) is the leading cause of death by female malignancy tumors, and the morbidity is increasing in urban areas each year [1, 2]. With advances in multidiscipline therapies including immunotherapy, the prognosis of patients with BRCA has improved dramatically. However, due to the significant variability in tumor heterogeneity, almost 62,667 patients died of BRCA in 2018 [3].

Immunotherapy is a promising strategy for cancer therapy that has a significant survival benefit in some BRCA patients. Immune checkpoint inhibitors such as anti-PD-1 and anti-PD-L1 that were approved as therapeutics for some malignant tumors have participated in various clinical trials, but only some patients responded well [4]. Selecting BRCA patients who will respond to immunotherapy is a critical topic right now.

The tumor microenvironment (TME) consists of tumor cells, immune cells, fibroblasts, extracellular matrix, chemokines, cytokines, etc. However, the immune and stromal cells in the TME are the primary nontumor components [5, 6]. Research on the TME demonstrates that it can downregulate the immune response to cancer therapy, which reduces the infiltration of dendritic cells and inhibits effector T cell activation [7, 8]. However, which factors regulate the components of the TME and influence the immune response to immunotherapy is unclear. Patients with melanoma, colon, or lung cancer along with a high tumor mutational burden (TMB) could benefit from immunotherapy [9–11]. However, the association between the TMB and tumor immunogenicity in BRCA patients is also unclear. Therefore, understanding the relationship between prognosis, the TME, and the TMB is crucial to identifying potentially effective immunotherapies.

Advances in bioinformatics and machine learning have enabled the widespread screening of cancer therapy biomarkers using high-throughput sequencing data [12]. The weighted gene coexpression network analysis (WGCNA) performed on genetic clusters and constructed coexpression modules provides the relationship between genes and modules, enabling an association between modules and the phenotypic traits of tumors [13, 14]. Therefore, we have the tools to identify TME-related genes related to the phenotypic traits of tumors for further study.

Overall survival (OS) is an appropriate endpoint for many clinical studies, especially for research on glioblastoma multiform [15]. However, for studies involving the least aggressive breast cancer subtype, luminal A, the progression-free interval (PFI) is suitable [16]. After integrating TCGA Pan-Cancer clinical database, Liu et al. [16] recommended OS and the PFI as the best endpoint events for TCGA analysis. In our study, we used both OS and the PFI as endpoints for further study of BRCA patients.

In the present study, we adapted bioinformatics and machine learning to construct a nomogram to predict the survival probability of BRCA patients and screening hub gene related with TME, which could be a new biomarker for selecting patients who might be a more likely response to immune checkpoint blockade therapies.

## 2. Materials and Methods

### 2.1. Data Source

**Figure 1:**
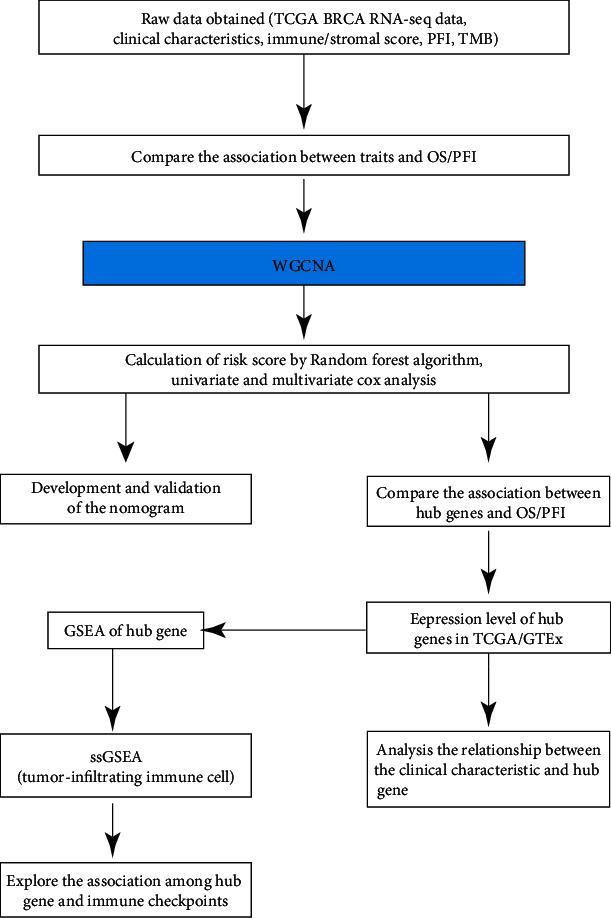
A schematic of the workflow for this study.

The RNA-Seq data and related clinical phenotypes were downloaded from TCGA database (http://cancergenome.nih.gov/). The PFI information was obtained from a previous study [17]. The immune score and stromal score of breast cancer samples were downloaded from MDACC (MD Anderson Cancer Center). The TMB data were obtained from the “tmb_data” source for the R package “UCSCXenaShiny.” Hallmark gene sets applied to the gene set enrichment analysis (GSEA) were sourced from the “msigdbr” package. Incomplete TNM stage samples were excluded, and 940 breast cancer samples were screened for further study. However, only 898 matched samples had PFI information available, which was required for a clinical study. Potential mRNA sequences were selected based on an RNA-Seq expression level greater than zero in at least half of the analyzed BRCA tissues (see Figure 1 for a workflow schematic).

### 2.2. Construction of the Weighted Gene Coexpression Network and Screening for Hub Genes

The R package “WCGNA” was used for the gene coexpression network analysis [18]. We set 200 as the height cut-off for the sample clustering analysis and log-transformed (log(count+1)) the RNA-Seq count value for further gene clustering analysis. We next calculated the optimal soft threshold for the adjacency computation. Twelve modules were screened for the WGCNA package one-step process. Gene significance (GS) was defined as the correlation between each trait and the gene expression level. The module membership (MM) was defined as the correlation between each module Eigengene (ME) and the gene expression level [13, 14]. The criteria for screening genes in relevant modules were a MM > 0.8 and a GS > 0.6 (*p* < 0.05). We also applied univariate and random forest analyses to screen for hub genes.

### 2.3. Construction and Calibration of the Nomogram

First, the stepwise multivariate Cox regression analysis was performed to construct the risk score formula, and the R package “survival” was applied (a model was chosen by AIC using a stepwise model selection) [3, 19, 20]. The following four-gene signature formula was constructed based on gene expression levels and coefficients (*β*):

(1) $$\begin{array}{c} \text{ risk score}=\overset{N}{\underset{i=1}{\sum }}Exp_{i}\times \beta_{i}. \end{array}$$

Second, the risk score was stratified into high-risk and low-risk groups based on the median. The risk score, M stage, N stage, tumor size, TNM stage, TMB, immune score, and age were selected as variables to construct the nomogram with the R package “rms.” We used the decision curve analysis (DCA), an emerging method for predicting the effectiveness of a model, to evaluate the discrimination of our nomogram [21]. The bootstrap method, a fast-development method based on random sampling with replacement, was used to internally validate the nomogram.

### 2.4. Hub Gene Pathway Enrichment Analysis and Correlation with Tumor-Infiltrating Immune Cells

We made a gene list to rank the correlation between a hub gene and others. Hallmark gene sets from the R package “msigdbr” were applied. A gene set enrichment analysis (GSEA) was then conducted with the “clusterProfiler” package [22]. We also performed a single-sample GSEA (ssGSEA) to identify the score of tumor-infiltrating immune cells in each sample using the “GSVA” package [23]. We next calculated the differential expression and correlation of tumor-infiltrating immune cells with the hub genes. We used the R package “circlize” to visualize the association between the hub genes and common immune checkpoint inhibitors.

### 2.5. Statistical Analysis

The Mann-Whitney *U*-test was used to compare the relationship of continuous variables in two groups; otherwise, the Kruskal-Wallis *H*-test was used. If the variables were categorical, the *χ*^2^ test (or Fisher's exact test) was applied. All data and figures were analyzed and plotted with R (version 3.6.3).

## 3. Results

### 3.1. Association between the TME, the TMB, and Prognosis in BRCA Patients

**Figure 2:**
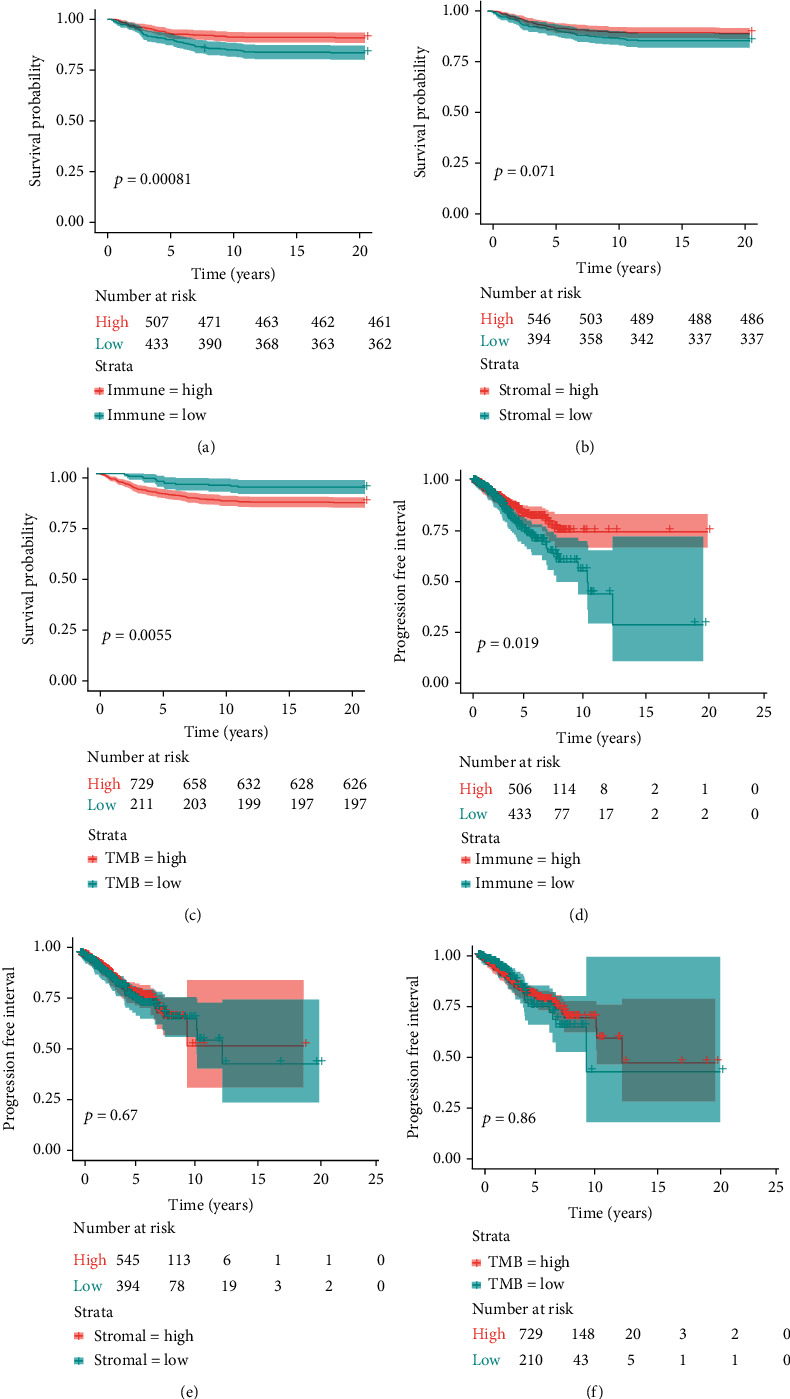
Immune score, stromal score, and TMB are associated with OS and the PFI. The K-M analysis and log-rank test were performed.

**Figure 3:**
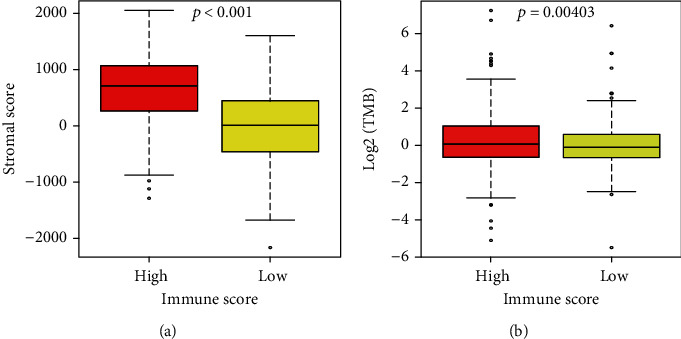
Correlation of the immune score with the stromal score and TMB.

The immune scores were divided into high-immune and low-immune score groups according to the optimal cut-off value. The K-M survival analysis and log-rank test were performed to identify the relationship between either OS or the PFI with the immune score. The survival probability of the high-immune score group was significantly higher than the low-immune score group, with a *p* value of 0.0028 (Figure 2(a)). The probability of a progression-free interval was also significantly higher for the high-immune score group than the low-immune score group (*p* value = 0.0084) (Figure 2(d)). Similarly, the stromal score and the TMB were classified into high-stromal and low-stromal score groups and high-TMB and low-TMB groups, respectively. The K-M survival analysis and log-rank test demonstrated that patients with a high-stromal score had no statistical difference with either OS (Figure 2(b)) or the PFI (Figure 2(e)) compared to patients with a low-stromal score. While patients with high-TMB have a lower survival probability than patients with low-TMB (*p* value = 0.0055) (Figure 2(c)), patients with high-TMB had no statistical difference with PFI compared to patients with low-TMB (*p* value = 0.86) (Figure 2(f)). Patients in the high-immune score group had a significantly higher stromal score and TMB than patients in the low-immune score group (Figure 3).

### 3.2. Construction of the WGCNA and Identification of Corresponding Modules

**Figure 4:**
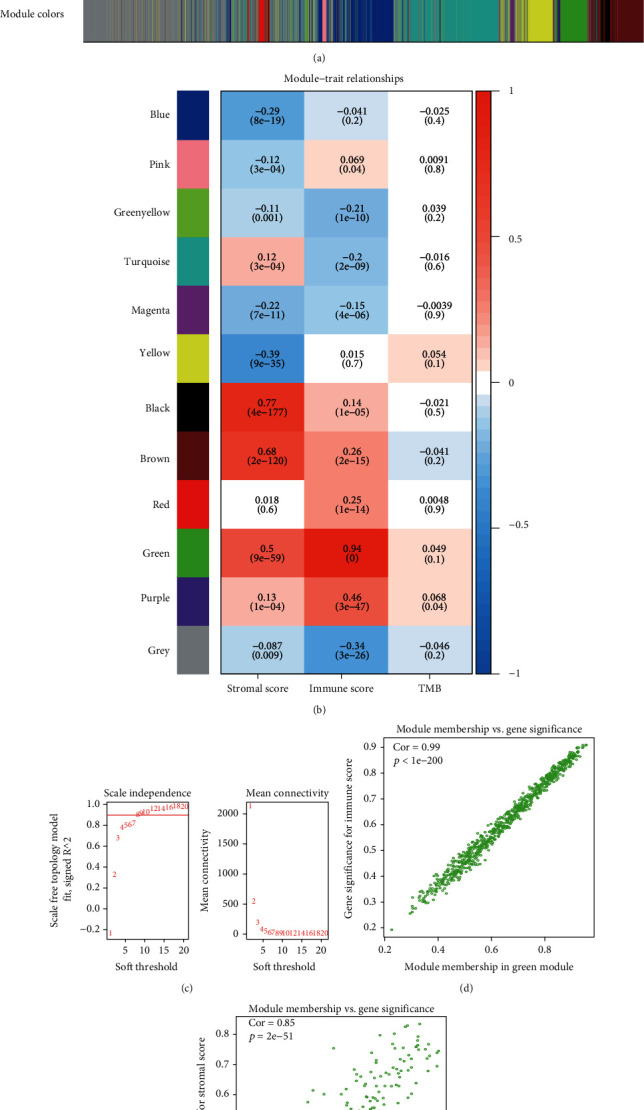
Construction of the WGCNA. (a) Cluster dendrogram. Each color represents a module, and branches represent genes. (b) Relationship between each module and trait. (c) Soft-threshold selection. (d) Association between MM in the green module and GS with immune scores. (e) Association between MM in the black module and GS with immune scores. MM: module membership. GS: gene significance.

Setting the criterion of protein-coding genes expressed in at least half of BRCA tissues, a total of 13721 mRNA sequences were screened for the WGCNA. The screen identified 12 modules (Figure 4(a)). The association between modules and traits was constructed and identified correlations between the green module and immune score (0.94) and the black module and stromal-score (0.77). Both *p* values were less than 0.001 (Figure 4(b)). In the present study, eight was defined as a set point for the soft-threshold power, and the related scale-free topology index was 0.9 (Figure 4(c)). Therefore, we further analyzed the green and black modules. The relationships between GS and MM were identified for both the green and black modules; the correlation was 0.99 and 0.85, respectively (*p* values < 0.001) (Figures 4(d) and 4(e)). In the green and black modules, 753 and 180 mRNA sequences were identified, respectively. We defined the cut-off as a MM > 0.8 and a GS > 0.6, narrowing our results for further study to 191 and 40 mRNA sequences in the green and black modules, respectively.

### 3.3. Calculation of the Risk Score by Random Forest, Univariate, and Multivariate Cox Analysis

**Figure 5:**
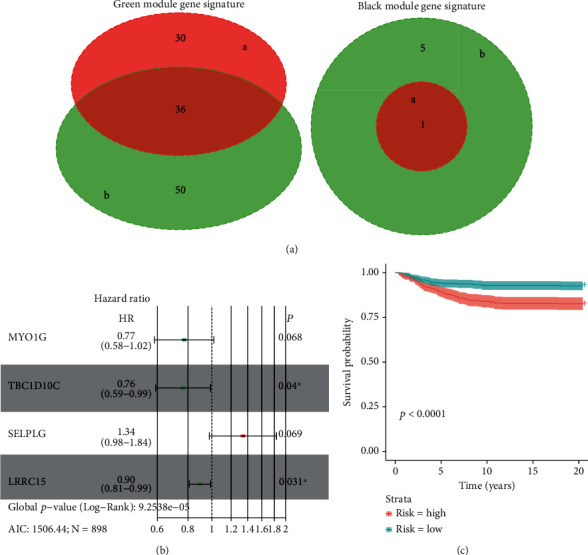
Construction of the risk score formula. (a) Venn diagram of hub genes from the random forest and univariate Cox analyses of the green and black modules. (a) Random forest. (b) Univariate Cox analysis. (b) Forest plot of the risk score model. P: *p* value; HR: hazard ratio; *N*: sample number; AIC: Akaike information criterion. (c) Correlation between the risk score and survival probability.

Random forest was performed for a survival analysis of the 191 mRNA sequences from the green module and 40 mRNA sequences from the black module, respectively. A univariate Cox analysis was used to examine the relationships between the expression of the 191 mRNA sequences in the green module, and the expression of the 40 mRNA sequences in the black module, with OS. Based on these results, 37 mRNA sequences (Figure 5(a)) were selected for a stepwise multivariate Cox analysis. In addition, a four-mRNA risk score formula was established (Figure 5(b)). Risk score = (−0.259 × expression level of^“^MYO1G^”^) + (−0.27 × expression level of^“^TBC1D10C^”^) + (0.292 × expression level of^“^SELPLG^”^) + (−0.11 × expression level of^“^LRRC15^”^). Based on the median value, the risk score was classified into high-risk and low-risk groups. A K-M survival analysis and log-rank test demonstrated that the high-risk group had a lower probability of survival than the low-risk group (*p* value < 0.001) (Figure 5(c)).

### 3.4. Development of the Nomogram for Predicting Survival Probability

**Figure 6:**
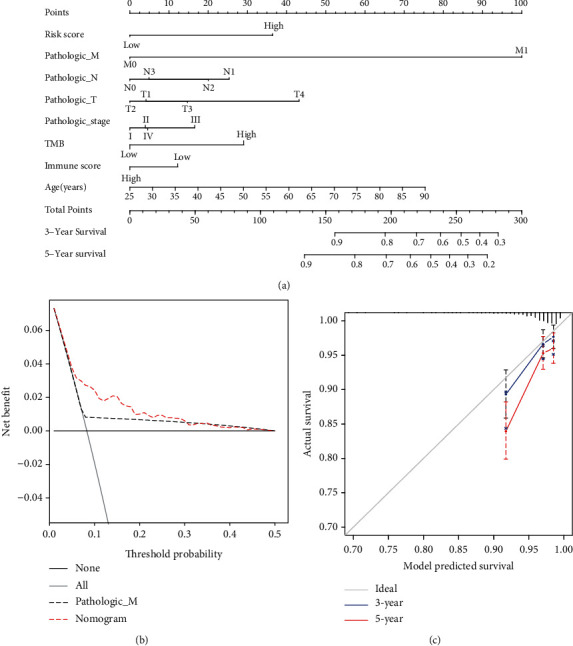
Construction and validation of the nomogram. (a) Nomogram for predicting the probability of 3- and 5-year OS in BRCA patients. (b) DCA of the nomogram OS prediction. (c) Calibration curve of the nomogram. The gray line indicates the ideal plot for the calibration curve. 3-year: 3-year OS predicted probability by nomogram. 5-year: 5-year OS predicted probability by nomogram. The bootstrap method was applied.

A multivariate Cox regression was performed to construct the prediction model (Figure 6(a)). The nomogram illustrates that the M stage made a robust contribution to the prediction of survival probability, followed by age, the tumor size, and the risk score. The score for each variable subtype was presented on a point scale. By calculating the total score and identifying it on the scale for the total possible points, we can easily obtain the survival probability of a patient. The DCA (decision curve analysis) is an emerging method to evaluate the discrimination of a predictive model [21]; it is widely used in many top journals, such as BMJ, JAMA, and NATURE. In our study, DCA to evaluate the predictability of our model demonstrated that the net benefit of our nomogram was significantly higher than others (Figure 6(b)). The *C*-index for the nomogram was 0.713, and the calibration curve demonstrated good performance by the nomogram (Figure 6(c)).

### 3.5. Relationship between Four TME Genes and BRCA Endpoints

**Figure 7:**
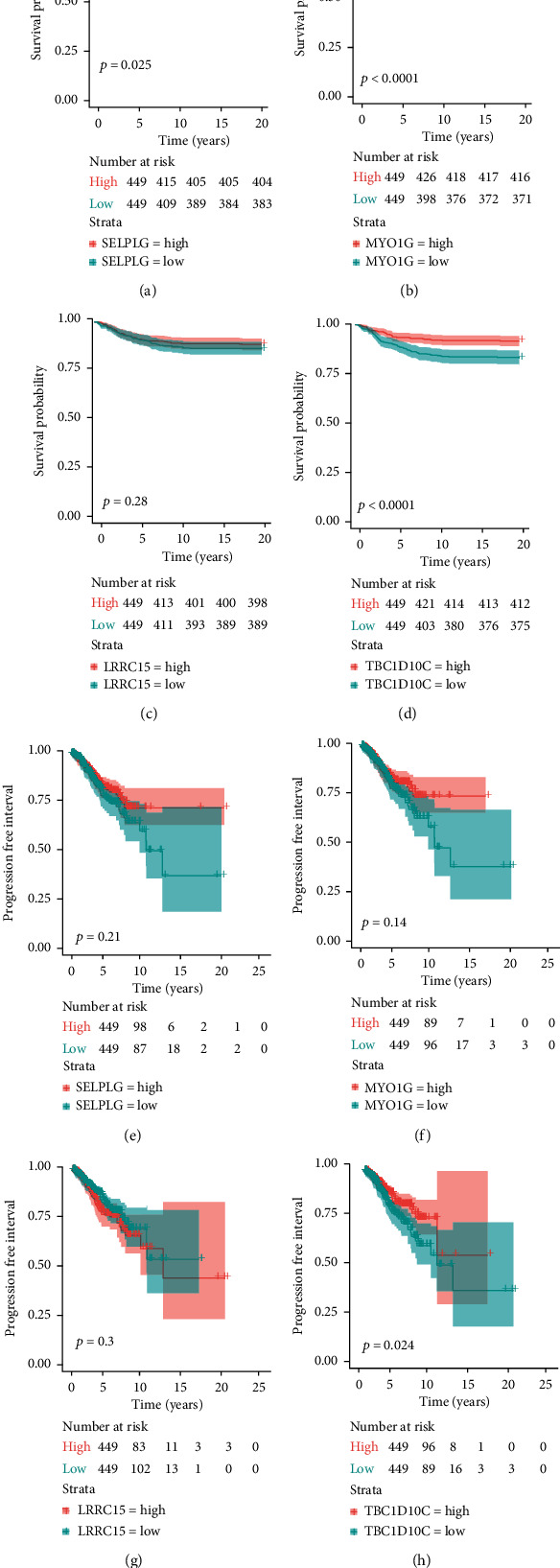
Correlation between all four hub genes and the BRCA endpoints: OS and PFI.

**Figure 8:**
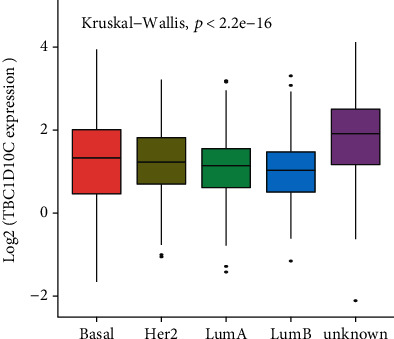
Distribution of TBC1D10C expression of breast cancer subtypes. The box plot shows that there is a significant association between breast cancer subtypes and the level of TBC1D10C expression.

We divided four genes (MYO1G, TBC1D10C, SELPLG, and LRRC15) into high and low groups based on the median expression value. The K-M analysis and log-rank test were applied. SELPLG and MYO1G expression had statistically significant correlations with OS in both the high and low expression groups, but not with the PFI (Figures 7(a), 7(b), 7(e), and 7(f)). The differential expression of LRRC15 was not correlated with either OS or the PFI (Figures 7(c) and 7(g)). The TBC1D10C high-expression group had a higher positive association with OS and the PFI than the low-expression group (Figures 7(d) and 7(h)). These data demonstrated that TBC1D10C is a protective marker. Meanwhile, we found there is a significant association between breast cancer subtypes and the level of TBC1D10C expression (*p* < 0.0001) (Figure 8).

**Figure 9:**
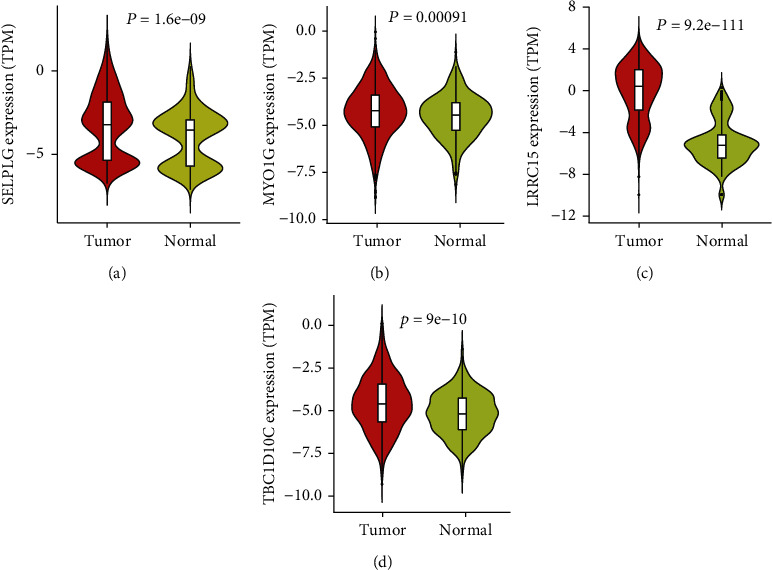
Differential expression of four hub genes between tumor and normal tissues. The tumor sample originated from TCGA database. The normal sample was downloaded from the GTEx database.

We next calculated the differential expression of all four genes in tumor tissue from TCGA database and normal tissue from the GTEx database. These data indicated that all four genes were more highly expressed in tumor tissues than in normal tissue (Figure 9).

### 3.6. Enrichment Pathway Analysis of TBC1D10C

**Figure 10:**
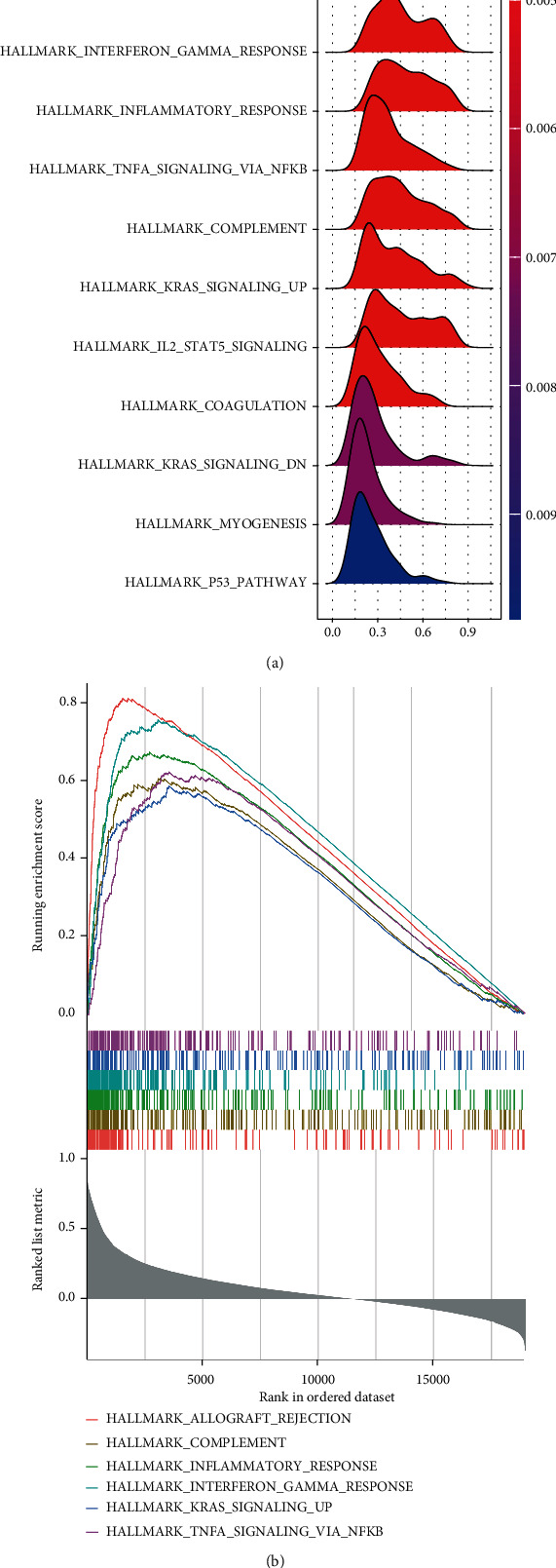
Enrichment pathway analysis of TBC1D10C. (a) The gene set was enriched in 11 pathways. (b) Top 6 scoring enrichment pathways.

Considering the differential prognosis for differential expression of TBC1D10C, we made a gene list to rank the correlation between TBC1D10C and other genes. GSEA indicated that it primarily correlated with, and thereby might be involved in, the allograft rejection, interferon-gamma response, inflammatory response, K-Ras signaling, TNFA signaling via the NFKB, and complement pathways (Figure 10(a)). The top 6 of the high enrichment score pathway is displayed in Figure 10(b).

### 3.7. Tumor-Infiltrating Immune Cells and Immune Checkpoints Correlation with TBC1D10C in BRCA Patients

**Figure 11:**
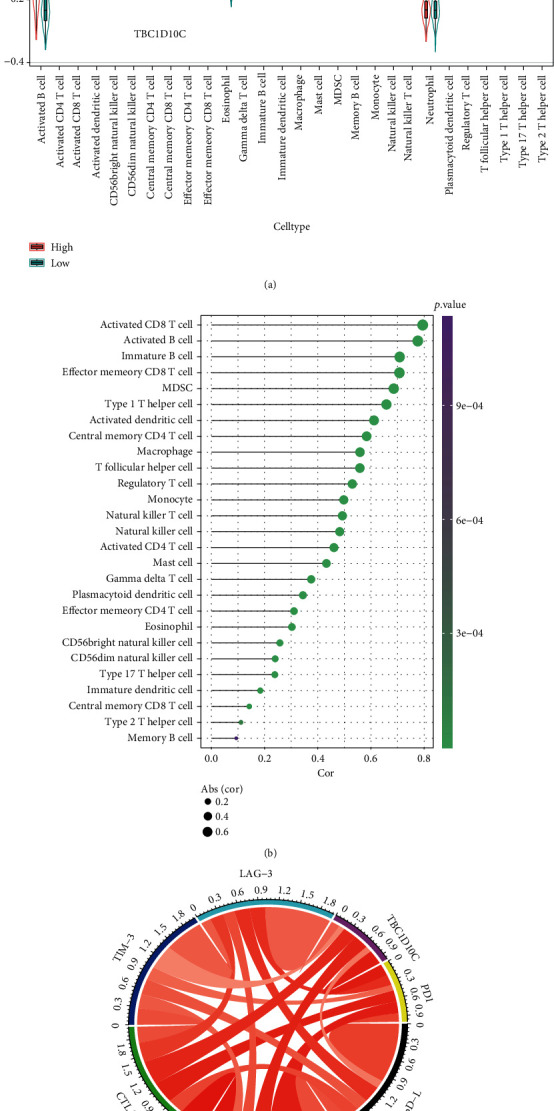
Correlation between TBC1D10C and either tumor-infiltrating immune cells or immune checkpoints. (a) 28 tumor-infiltrating immune cells express levels between the high and low groups of TBC1D10C. (b) Correlation between TBC1D10C and tumor-infiltrating immune cells (*p* < 0.05). 4(c) Association between TBC1D10C and immune checkpoints.

We next evaluated the relationship of TBC1D10C expression levels with tumor-infiltrating immune cells in the breast cancer microenvironment using ssGSEA (Figure 11(a)). Only 3 of 28 immune cell types (central memory CD8 T cells, memory B cells, and neutrophils) that were differentially expressed had no statistical significance. The tumor-infiltrating immune cells, e.g., activated CD8 T cells and activated B cells, had a robust correlation with TBC1D10C (*p* value < 0.05) (Figure 11(b)).

Immune checkpoint blockade therapies are emerging and effective strategies for treating cancer [24]. We next explored the associations between TBC1D10C and immune checkpoints including PD1, PD-L1, TIGIT, CTLA-4, TIM-3, and LAG-3. The chord chart indicated that TBC1D10C had a robust positive correlation with PD1, TIGIT, and CTLA-4 (Figure 11(c)).

We further analyzed the relationship between the clinical characteristics of BRCA patients and TBC1D10C expression levels. These data indicated that the differential expression of TBC1D10C had a statistically significant difference according to the PAM50 subtype, M stage, N stage, stromal score, and immune score (Table 1).

## 4. Discussion

Recently, the relationship between the TME and immune therapy has become an increasingly hot issue. Differential quantities of tumor-infiltrating immune cells result in diverse responses to immunotherapy [25, 26]. We applied the immune and stromal scores downloaded from MD, which were calculated by the ESTIMATE algorithm [27], to represent the status of the TME. We next performed WGCNA to identify modules correlated with immune and stromal scores. Finally, four hub genes (MYO1G, TBC1D10C, SELPLG, and LRRC15) that correlated with the TME were selected to construct a nomogram to predict the survival probability using a univariate and multivariate Cox regression and random forest. The only gene to have a clear correlation with both OS and the PFI was TBC1D10C. We further focused on the function of TBC1D10C and its association with tumor-infiltrating immune cells and common immune checkpoints.

Overall survival (OS) is a vital endpoint with less ambiguity in defining an overall survival event. However, OS as an endpoint can attenuate a clinical study since noncancer death also qualifies as an endpoint event [28, 29]. The progression-free interval (PFI), characterized as the minimum follow-up time needed, is also used in many clinical trials [28], as patients with disease recurrence or progression usually have a long lifespan. In the present study, both OS and the PFI as endpoint events were included in our study to screen OS-related and PFI-related hub genes. Only the gene TBC1D10C had a clear correlation with both OS and the PFI. This indicates that TBC1D10C may be a potential biomarker in cancer recurrence and progression.

We also found that TBC1D10C is an immune-related gene using the Immport database (https://www.immport.org) [30]. TBC1D10C, also known as Carabin or EPI64C, is overexpressed in blood leukocytes and the spleen and negatively regulates the NF-*κ*B signaling pathway via activity as a Ras GTPase-activating protein [31]. Moreover, TBC1D10C physically interacts with CaN T cells and H-Ras in addition to inhibiting Ras/MAPK signaling [31, 32]. Our GSEA indicated that TBC1D10C6 regulated the K-Ras-signaling pathway, which is consistent with the existing research [33]. Moreover, ssGSEA revealed that TBC1D10C has a high positive correlation with B cells and T cells, which is consistent with the research of Jiang et al. [31] but contradicts Schickel et al. [32]. The opposing results may be due to study differences; one focused on lymphoid disease while the other was interested in myocardial disease.

TMB is a vital promising biomarker that plays a key role in predicting the response to immune checkpoint blockade therapy in several cancer types [34]; however, few studies have focused on the significance of TMB in BRCA. Mei et al. [35] revealed that high TMBs occur at a low frequency in BRCA. Narang et al. [36] demonstrated that triple-negative breast cancers have the highest TMB, followed by the HER2-positive subtype. In our study, higher TMBs have a lower survival probability than lower TMB patients but have no statistical significance with the PFI. Conflicting results may be due to high tumor heterogeneity in BRCA and varying therapies.

Tumor immune checkpoint inhibitors have been proven as effective therapies for many malignant tumors. BRCA, however, has highly heterogeneous tumors, preventing many patients from benefiting from immunotherapy [37]. Misidentifying patients who cannot respond to immunotherapy is potentially fatal. TBC1D10C was highly correlated with three common immune checkpoints (PD-1, CTLA-4, and TIGIT) in our study and associated with OS and the PFI. These data suggest that TBC1D10C may be a new immune checkpoint.

There are still many gaps left to be understood. First, we only conducted an internal validation of the nomogram with bootstrapping. However, the lack of external validation limits the generalization of our results. Second, TCB1D10C was overexpressed in tumor tissue from TCGA database compared with normal tissue from the GTEx database; however, studies, such as RT-PCR and Western blot, are needed to validate TCB1D10C expression. Third, the function of TCB1D10C and the mechanism by which it regulates the K-Ras signaling pathway needs further exploration.

## 5. Conclusions

By performing WGCNA and machine learning, we constructed a TME-related gene signature model for predicting the survival probability of BRCA patients. Subsequently, we identified a hub gene, TBC1D10C, that correlated with OS and the PFI and had a high positive association with tumor-infiltrating immune cells and three common immune checkpoints (PD-1, CTLA-4, and TIGIT). TBC1D10C may be a new biomarker for identifying patients that would benefit from immunotherapy.

## Figures and Tables

**Table 1:** Clinical characteristic of patients between high and low expressions of TBC1D10C.

	Overall	TBC1D10C		*p* value
		High expression	Low expression	
*n*	898	449	449	
ER_Status (%)				0.065
Negative	196 (21.8)	112 (24.9)	84 (18.7)	
Positive	658 (73.3)	314 (69.9)	344 (76.6)	
Unknown	44 (4.9)	23 (5.1)	21 (4.7)	
PR_Status (%)				0.954
Negative	280 (31.2)	142 (31.6)	138 (30.7)	
Positive	571 (63.6)	284 (63.3)	287 (63.9)	
Unknown	47 (5.2)	23 (5.1)	24 (5.3)	
HER2_Status (%)				0.007
Negative	674 (75.1)	323 (71.9)	351 (78.2)	
Positive	122 (13.6)	60 (13.4)	62 (13.8)	
Unknown	102 (11.4)	66 (14.7)	36 (8.0)	
PAM50 subtype (%)				<0.001
Basal	118 (13.1)	61 (13.6)	57 (12.7)	
Her2	62 (6.9)	30 (6.7)	32 (7.1)	
LumA	362 (40.3)	160 (35.6)	202 (45.0)	
LumB	166 (18.5)	59 (13.1)	107 (23.8)	
Unknown	190 (21.2)	139 (31.0)	51 (11.4)	
M stage (%)				0.004
M0	882 (98.2)	447 (99.6)	435 (96.9)	
M1	16 (1.8)	2 (0.4)	14 (3.1)	
N stage (%)				0.013
N0	413 (46.0)	212 (47.2)	201 (44.8)	
N1	304 (33.9)	142 (31.6)	162 (36.1)	
N2	119 (13.3)	53 (11.8)	66 (14.7)	
N3	62 (6.9)	42 (9.4)	20 (4.5)	
T stage (%)				0.09
T1	236 (26.3)	128 (28.5)	108 (24.1)	
T2	532 (59.2)	259 (57.7)	273 (60.8)	
T3	101 (11.2)	53 (11.8)	48 (10.7)	
T4	29 (3.2)	9 (2.0)	20 (4.5)	
TNM stage (%)				0.051
I	157 (17.5)	80 (17.8)	77 (17.1)	
II	526 (58.6)	269 (59.9)	257 (57.2)	
III	201 (22.4)	98 (21.8)	103 (22.9)	
IV	14 (1.6)	2 (0.4)	12 (2.7)	
Age [IQR]	58.00 [48.00, 67.00]	58.00 [49.00, 66.00]	58.00 [48.00, 67.00]	0.92
TMB [IQR]	0.97 [0.63, 1.78]	0.93 [0.58, 1.89]	1.01 [0.66, 1.71]	0.412
Stromal_score [IQR]	414.52 [-163.90, 838.41]	554.52 [90.80, 967.12]	204.14 [-339.70, 700.80]	<0.001
Immune_score [IQR]	121.41 [-362.02, 691.89]	638.86 [170.02, 1224.44]	-287.32 [-614.74, 72.88]	<0.001

ER: estrogen receptor; PR: progesterone receptor; HER2: human epithelial growth factor receptor 2. IQR: interquartile range. TMB: tumor mutational burden. *n*: sample numbers.

## Data Availability

The RNA-Seq data and related clinical phenotypes were downloaded from TCGA database (http://cancergenome.nih.gov/). The PFI information was obtained from this study (doi:10.1016/j.celrep.2016.12.019). The immune score and stromal score of breast cancer samples were downloaded from MDACC (MD Anderson Cancer Center). The TMB data were obtained from the “tmb_data” source for the R package “UCSCXenaShiny.”
